# Effects of Pain From Atopic Dermatitis: Interview and Focus Group Study With Patients and Their Families

**DOI:** 10.2196/29826

**Published:** 2021-10-04

**Authors:** Ashley M Snyder, Vanina L Taliercio, Adelheid U Brandenberger, Bianca E Rich, Lisa B Webber, Abram P Beshay, Joshua E Biber, Rachel Hess, Jamie L W Rhoads, Aaron M Secrest

**Affiliations:** 1 Department of Dermatology University of Utah Salt Lake City, UT United States; 2 Department of Population Health Sciences University of Utah Salt Lake City, UT United States; 3 School of Medicine University of Utah Salt Lake City, UT United States; 4 Department of Internal Medicine University of Utah Salt Lake City, UT United States

**Keywords:** atopic dermatitis, eczema, pain, qualitative research, quality of life

## Abstract

**Background:**

Pain is an underappreciated symptom of atopic dermatitis that can affect the health-related quality of life (HRQL) of patients.

**Objective:**

The aim of this study is to understand the effect of pain on patients with atopic dermatitis and their family members and to recognize how this symptom affects HRQL.

**Methods:**

We conducted focus groups and interviews with patients with atopic dermatitis and their family members. Researchers independently coded the transcripts and reached a consensus on the major themes.

**Results:**

A total of 33 adult participants, consisting of 21 patients with atopic dermatitis (median age 47 years, range 22-77) and 12 family members (median age 50, range 22-72), attended either focus groups (23/33, 70%) or interviews (10/33, 30%), where we assessed their experiences of pain. Four themes emerged in our study. Itchiness and pain can be intertwined: pain was often caused by or otherwise associated with itchiness and could result from open sores and excoriated skin. Characteristics of pain: pain was most often described as burning. Other descriptors included mild, persistent discomfort; stinging; and stabbing. Effects of pain: pain negatively affected various aspects of daily life, including choice of clothing, sleep, social activities, and relationships. The location of painful areas could also limit physical activity, including sex. Pain management: pain from atopic dermatitis could be managed to varying degrees with different over-the-counter and prescription treatments. Systemic agents that cleared the skin also resolved the pain associated with atopic dermatitis.

**Conclusions:**

Pain can be a significant factor in the HRQL of patients with atopic dermatitis and should be considered by clinicians when caring for patients with atopic dermatitis.

## Introduction

### Background

Atopic dermatitis is a chronic inflammatory skin disease with a highly symptomatic clinical course [1]. The hallmark symptom of atopic dermatitis is itch, or pruritus [1]. However, pain has also been recognized as an important and highly prevalent symptom in patients with atopic dermatitis [2]. An international web-based survey of 1111 patients with atopic dermatitis from 34 countries showed that pain was the second most common symptom of atopic dermatitis after itchiness [3]. A prospective practice-based study found that over 40% of patients with atopic dermatitis experienced some level of pain [4], and a more recent cross-sectional study found that 61% of participants reported experiencing pain from atopic dermatitis [2].

Several studies have analyzed the effect of chronic pain on the lives of patients, highlighting the strong correlation between pain and the deterioration of health-related quality of life (HRQL) [5-8], and atopic dermatitis is no exception. Some patients with atopic dermatitis find that their pain is related to scratching, fissures on the skin, inflamed red skin, or burning from creams or ointments [2]. Pain from atopic dermatitis can have a negative impact on the ability of patients to shop, make clothing decisions, and maintain relationships, among other aspects of daily life [4]. Moreover, although pain is not universally experienced, one study found that the proportion of patients with moderate or severe atopic dermatitis who reported pain as the most burdensome symptom was more than six times the proportion of patients with mild atopic dermatitis who reported the same [9].

### Objective

Due to the time constraints of clinical practice, dermatologists are often unable to gain an in-depth understanding of all symptoms that the patient has and how they affect the HRQL of a patient. Dermatologists’ assessment of the frequency of primary symptoms of a patient (often itchiness with atopic dermatitis) may not accurately reflect the perspectives of the patient. For diseases in which symptoms become debilitating, the primary goal of treatment is symptom control. Failure to understand how debilitating symptoms are for patients may leave them undertreated. We aimed to document the qualitative experience of atopic dermatitis from the perspectives of patients and their families, specifically focusing on the understudied symptoms of pain with atopic dermatitis.

## Methods

### Study Design

We conducted a qualitative study using focus groups and interviews to understand the effect of pain on HRQL in patients with atopic dermatitis and their family members. Our study was approved by the University of Utah institutional review board (#98441).

### Participants and Setting

Participants were recruited either by recommendation from their dermatologist or by identifying patients with a diagnosis of atopic dermatitis in the electronic medical record with an upcoming appointment and having the study coordinator ask them about participating in the study at the time of the appointment. To be included, patients had to be aged ≥18 years, speak English, and have atopic dermatitis diagnosed by a University of Utah dermatologist. Family members had to be a partner or first-degree relative of a patient with atopic dermatitis, be aged ≥18 years, and speak English.

We attempted to recruit a sufficiently large sample to achieve thematic saturation. In total, 21 patients and 12 family members participated in the focus groups or interviews. Each participant provided demographic data via a written survey and written informed consent before the focus group or interview. One family member also had atopic dermatitis, so they contributed data from both family member and patient perspectives.

### Data Collection and Analysis

Semistructured interview guides with open-ended questions were developed regarding experiences with itchiness, pain, sleep quality, and personal relationships because of atopic dermatitis (Textbox 1). The principle of theory-driven qualitative research was used, including basing themes and interview questions on theoretical considerations, expert discussions, and an extensive literature review. Investigators used an integrative model of patient-centeredness to guide the development of guidelines [10,11]. Interviews lasted about 15-35 minutes, and focus groups lasted about 60-70 minutes. Meetings were audiorecorded and transcribed verbatim, though some utterances were left out if not considered important for meaning, for example, “mm-hmm.”

**Interview guide questions about pain from atopic dermatitis (eczema).**

**Interview guide questions**

Please tell me more about the pain associated with the eczema.

- What words would you use to describe the pain of the eczema?
  Prompts: burning, stinging, pins and needles, aching, stabbing, tingling.
- What time of day is the eczema most painful?
- What are things that cause pain with eczema?
  Prompts: scratching too much, putting creams/lotions on your eczema
- What helps when the pain is worst, what provides relief?
- How does eczema’s pain affect your sleep? The sleep of your significant other?
  How often is pain affecting sleep? How does it affect your bedroom relationship?
- How does eczema’s pain affect your mood? The mood of your loved ones?
- How does pain from eczema affect relationships?
- What other areas of your life are affected by the pain of eczema?

A thematic analysis approach was used to assess transcripts [12,13]. Two investigators (AM Snyder and VLT) with previous training in qualitative methods assessed several transcripts for themes and ideas for an initial codebook, and themes evolved as they continued to code all the transcripts and analyzed the final set of codes. The two investigators reached a consensus on the major themes. Although no changes were made to the meaning of the quotes, it should be noted that minor aspects of some quotes (eg, punctuation and grammatical errors) were changed to account for errors during transcription. For the presentation of quotes, words inserted into quotes or replacing specific words to help convey meaning are shown in brackets; descriptions of actions during the interview or focus group are italicized and shown in brackets; and any text left out of a quote, whether to shorten the quote or replace the text where there was uncertainty about the transcription, was replaced by bracketed ellipses. NVivo version 12 (QSR International) was used in assessing the codes. STATA version 16 (StataCorp) was used to calculate the demographic statistics.

## Results

### Demographic Characteristics

**Figure 1:**
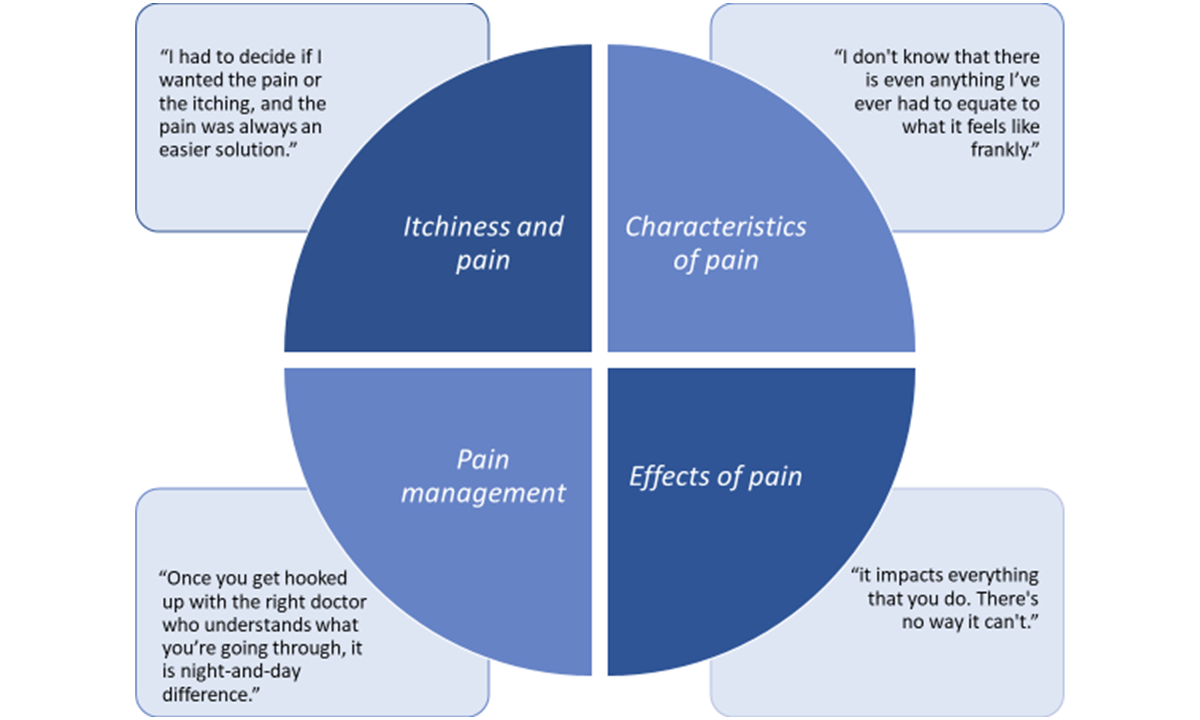
Four main themes regarding pain were elucidated from the transcripts of focus groups and interviews with patients with atopic dermatitis and their family members.

Participants attended either focus groups (23/33, 70%) or interviews (10/33, 30%), where we assessed the experiences of 21 patients with atopic dermatitis and 12 family members. Most participants identified as female and non-Hispanic White (Table 1). Four themes emerged that indicated that pain had a significant effect on HRQL (Figure 1). Not all participants reported pain, but for those who did, pain created many different sensations and a variety of effects on daily life.

**Table 1:** Demographic characteristics of patients and family members (N=33).

Characteristics			Patients (n=21)		Family members (n=12)
Age (years), median (range)			47 (22-77)		50 (22-72)
**Gender, n (%)**					
	Female	16 (76)		8 (67)	
	Male	5 (24)		4 (33)	
**Race and ethnicity, n (%)**					
	Non-Hispanic White	20 (95)		10 (83)	
	Other	1 (5)		2 (17)	
**Education,** **n (%)**					
	High school	3 (14)		2 (17)	
	College degree	7 (33)		6 (50)	
	Bachelor’s degree	7 (33)		3 (25)	
	Master’s degree or higher	3 (14)		1 (8)	
	Not reported	1 (5)		0 (0)	
**Marital status, n (%)**					
	Married or domestic partner	16 (76)		11 (92)	
	Single, widowed, or divorced	5 (24)		1 (8)	
**Employment status, n (%)**					
	Employed	11 (52)		10 (83)	
	Retired	4 (19)		2 (17)	
	Unable to work	1 (5)		0 (0)	
	Unemployed	4 (19)		0 (0)	
	Not reported	1 (5)		0 (0)	

### Theme 1: Itchiness and Pain

Scratching was a necessity for some patients with atopic dermatitis, and pain was sometimes an unfortunate consequence of scratching to try to relieve itchiness. The urge to provide relief from itchiness could be more powerful than the desire to avoid further consequences. This behavior of *inducing pain* led to physical consequences for some, such as open sores and denuded skin:

[That] is probably where the pain is coming from [...] he’s scratched it raw and then it, especially on the inner crooks of his elbows and stuff like where he’s bending them a lot, it just kind of opens those wounds back up.

Conversely, scratching was not always viewed as a negative as scratching could provide relief from the pain:

[Sometimes] you get the pain, but sometimes if you scratch it, now you have the sensation of scratching and now it’s not hurting like it did in that moment before the scratch. You jarred it up a little bit so your body can get a break from the pain, some relief.

Itchiness and pain were sometimes difficult to distinguish from each other. This *painful itch* phenomenon experienced by some patients appeared to be a significant component of the effects of the condition. One patient explained:

[It] doesn’t feel like it’s just itching on top of the skin; it itches down in the skin [...] if you rub it and scratch it, feels almost like a thrill going through your body but it’s a hurting thrill or [...] it’s like you’re having sex or something and you get that climax. It is like that but the only thing is it’s pain.

This pain, described as *coming from deep within*, might not be stopped simply by scratching an itch. Patients in our study generally struggled with itchiness, but coexistent pain worsened daily struggles with atopic dermatitis.

### Theme 2: Characteristics of Pain

Itchiness was not always a part of the pain sensation for patients; instead, some patients felt a burning sensation that could be just as irritating. A *burning pain* was a somewhat common description of the pain experienced:

[It’s] amazing how there aren’t any scars from it, because it’s like a fire. I mean it’s like you got burnt.

One patient explained:

There are times that my skin just feels like it’s on fire and there is nothing [that] will put it out.

Additional descriptors of pain included: *hot and burning, stabbing, dull, sharp, sunburn, sting,* needle-like, or even a persistent *discomfort* (Textbox 2). Some participants had difficulty describing their experience, and one patient expressed having trouble even talking about it:

Pain [...] is hard to talk to people about, because people don’t view [atopic dermatitis] in terms of pain.

**Example descriptions of pain from atopic dermatitis.**

**Descriptions of pain**

- Burning: “It is hot and burning. It’s true. And it’s like you just feel so helpless and nothing makes it feel better.”
- Discomfort: “I think...it’s not so much like the little bit of itchiness bothers me. It’s not so much that a little bit of pain that bothers me. It is more of the persistence of the two. You know what I mean? If I had it for a day and it went away, I’d be like, ‘Alright, that wasn’t so bad.’ Once it gets [to be], like, two to three weeks, then it’s like, ‘Alright, I’ve got to do something.’ They always ask me what’s the level of pain. I always felt that pain is not the right word for me. I feel like it’s more discomfort. I can sit and do my normal work and be bothered by it but not like in pain [...] just the persistence of the discomfort.”
- Dull: “there is just an overall dull pain that is never-ending.”
- Itching: “You will itch it even if it hurts to itch, because the itch feels way better than the pain does.”
- Needle-like: “because sometimes they would get infected, he would describe it as like, like needles, like poking.”
- Sharp: “I am glad you guys are focusing on the pain, because I feel like that is one aspect that is not even considered [...] I mean, it is just never stopping. It [is] like dull pain, sharp pain. Like you were saying, all the time.”
- Stabbing: “it is like a stabbing pain that radiates. If you think of like a reverse lightning bolt, there is one spot that hurts more than others, but it is also radiating to different areas—like a jab or a jolt. It feels like an itch, like a painful itch.”
- Sting: “When mine breaks out, I say it burns, but it’s not heat burn. It’s more probably a sting. But I always say burn, but when I think about it, it’s probably more like a sting.”
- Sunburn: “it rubbed and I couldn’t hardly walk. I couldn’t hardly stand it, and [...] I was achy. [...] You know how you feel when you get sunburnt? Your skin just burns. [...] It doesn’t go away.”

### Theme 3: Effects of Pain

When present, pain could result in various consequences for daily life. Patients with atopic dermatitis and their family members expressed having to be careful about their clothing choices. For example, having clothes touch affected skin could lead to pain:

[It] was mostly on his legs [...] the socks will really hurt.

This could lead to avoidance of certain clothes to avoid pain from atopic dermatitis:

When she gets these open sores [...] she could not wear bras or anything if it was in the sweat line.

Conversely, as one patient expressed, clothes that cover more skin could be a necessity:

[When] it’s so hot, I watch everyone else and they’ve got short-sleeved shirts. I’m all covered up, because the sun is so hard on my skin then. [...] I cannot wear sleeveless or short-sleeve clothes at all.

The pain of an atopic dermatitis flare-up can also affect daily activities. Pain was not always limited to one time of day: “my pain goes all day long.” However, the time of day was reported to make a difference for some and could particularly affect sleep:

It does hurt more when you lay down to go to sleep because I think you are starting to think about it more when you’re just lying there.

Sleep was also affected for some family members. One family member expressed concern over the consequences of their loved one’s pain during the night:

...when I know it’s flaring up, I might sleep a little bit lighter just because I don’t want him to wake up super, you know, bloody and in a ton a pain, and so I have a tendency to wake up a couple times a night and be like, “Hey, let’s put some lotion [on it].” But that’s really only when it’s flaring up really bad.

However, if the patient cannot control their reactions, family member comments are not helpful:

He will say, “Do not itch it. It is going to hurt.” But I can’t control it; it’s really itchy.

Daytime activities are also affected by atopic dermatitis–related pain. A hobby such as gardening became too painful to continue for a patient with atopic dermatitis:

My mom used to like gardening, but she doesn’t anymore because her hands are so affected by it. But she tries to wear gloves, but it still just, like, kills her.

However, as one patient expressed, other hobbies may then become favorable for their lack of resulting pain:

I read, because that’s one thing I can do in spite of the pain.

Showering can also be painful:

If he just gets out of the shower, he’ll say it feels like it burns.

The skin care regimen of a patient can be tedious and limited:

I use surgical soap as a body soap at this point. I miss pretty smells, just smelling pretty, smelling like a girl.

Warm environments can be problematic:

In the summertime just, the sweat. [...] She spent a lot of time indoors with the air condition on, but if we have to go out, or we go to the store and she starts sweating, you know, I can tell that she will get uncomfortable.

Furthermore, going out can be painful both physically and emotionally:

[It’s] depressing to go out. I’ll put on makeup. [...] I haven’t been able to wear eyeshadows or anything because my eyes are so sensitive [and they] swell up, and it’s caused me to have a depression, just the itching and burning.

Activities influenced specifically by pain were mentioned less often than those influenced by itchiness, but the range of effects was broad.

Similarly, social experiences can be affected by pain, and relationships can be strained by trying to care for pain in the affected patients. Physical relationships with a partner can be limited by pain from atopic dermatitis, and the location of the painful areas can limit sexual intimacy, as one patient explained:

It affected [...] our sex life, because I have sores all over my chest [...] It is painful [...] I am so self-conscious of it. [...] now this has been going on for eight years, so there’s been nothing in this area going on intimately, you know, with my husband.

For family members, the pain of atopic dermatitis can be difficult simply because they see the burden of the disease:

Every time she scratches, she’s talking about it hurting and wishes she could get something to stop it from scratching.

However, for family members, the effects of pain can be difficult to understand without knowing what the pain is like: “I can’t feel the pain.” However, the understanding of the family members of the effects may still be better than the understanding of others outside the family:

You pick up on it a lot faster if you’re living with them. We go over to friends’ houses, and they don’t understand it.

This lack of understanding, both from family members and others, can be frustrating for the patient:

The explaining to everyone what it is. You’re at the grocery store and you have a big cut on your hand, and people are like, “Did you get in a fight?” and you are like, “No!” Yeah, it’s just a crazy situation that is hard to explain. I wish they had groups for it because it is nice to talk to you [others in the focus group] because it is this connection that nobody else can understand, because even your partner doesn’t understand the exact level to which it affects you on a daily basis.

### Theme 4: Pain Management

Another important component was the use of over-the-counter and prescription treatments to control the symptoms. Prescription treatments could be very helpful in improving the HRQL of the patient:

I started the Dupixent and, oh my gosh, what a life-saver. [...] I can’t believe the difference.

One patient found cannabidiol helpful*:*

[She] has been using CBD oil and [...] that really has helped the pain and helped her sleep.

Steroids, such as triamcinolone, as well as over-the-counter treatments, such as acetaminophen and ibuprofen, were mentioned as ways to relieve pain. However, not all treatments work:

[Betamethasone] really doesn’t help. But I will put it on as soon as they pop out.

Some medications, such as pregabalin, were mentioned as being ineffective for atopic dermatitis–associated pain:

It kind of helps with these things [patient pointing to atopic dermatitis on hand]. But it wasn’t necessarily nerve pain.

One patient explained that it could also take time for a medication to start working:

It takes some time; I mean like when I was taking cyclosporine and it didn’t happen, like, overnight where it just stopped.

In addition to pain directly associated with atopic dermatitis flares, open wounds can become infected, requiring special treatment:

I think he scratches them to the point where they are open wounds and then they get a little infected, and so a lot of times we use a lot of Neosporin and stuff like that on his arms just, like, to keep the pain and everything at bay.

One patient expressed difficulty in discerning between atopic dermatitis–related pain and treatment-related pain:

I think certain ones are painful. [...] I’m trying to think if the creams cause the pain, and I don’t think creams cause the pain.

The pain for some patients was a direct result of medication use:

[He’s] been on steroids; it’s made his skin super thin [...], so he gets cuts all the time [...] he can’t even go get stitches anymore ‘cause his skin’s so thin it just tears it. [...] it makes him not want to do stuff, which is hard for me ‘cause I like to go out and do stuff and try new things and be active and he’s a little more hesitant because he’s just prone to getting hurt and sores and they take months to heal.

Finding clinicians who could significantly help was difficult for some participants. Patients with atopic dermatitis and their family members had a variety of experiences with clinicians when figuring out treatments for symptoms of atopic dermatitis—some positive and some negative.

## Discussion

### Principal Findings

Atopic dermatitis is known for its itchiness; however, our study found that focusing only on itchiness does not capture the full range of experiences of patients with atopic dermatitis and their family members. This study found that pain could coincide with itchiness, be difficult to describe, and influence all aspects of daily life for patients (and their family members). In addition, systemic agents could clear atopic dermatitis while also resolving the pain associated with this condition. The effects of pain from atopic dermatitis are relatively understudied but deserve further attention, both to identify the effects of pain on HRQL and to determine how to appropriately measure and potentially manage this symptom [14].

Increased frequency and intensity of pain from atopic dermatitis leads to worse HRQL, and for some, pain can affect relationships and numerous daily activities [2]. A study assessing the effects of pain from atopic dermatitis on patients and their caretakers found that 80% of participants had experienced effects on sleep, nearly as many (78%) experienced effects on leisure activities, and most (63%) experienced effects on other activities of daily living [15]. Although most comments in our study related to effects on HRQL were made about itchiness or atopic dermatitis more generally, we found that some patients experienced pain that affected the activities of daily living (eg, clothing choices, exercise, environmental exposures, and sexual relations) and could further affect overall HRQL. However, not all participants shared the same level of impact, likely due to differences in disease severity.

Some expressed having regained control over symptoms through medications. The use of systemic treatments, such as dupilumab, greatly improved both symptoms and HRQL outcomes for some participants, an observation supported by the literature [16]. However, not all systemic treatments share the same level of success [16], and prescribing systemic treatments must be carefully considered based on side effects, as was expressed by some participants in our study.

One of the most challenging aspects of pain is defining it. The root cause of pain from atopic dermatitis can be difficult to identify; one study found that about 17% of patients with atopic dermatitis thought pain was part of itchiness, whereas 72% thought it was part of both scratching and itchiness [4]. This is consistent with our results, as itchiness and pain overlapped for several patients. The two symptoms could be almost indistinguishable, and for some, scratching due to itchiness led to pain, although this trade-off was worth it when the pain felt better than an itch. The sensation of pain could be difficult to describe, underlining the problem that pain is a poorly understood and ill-defined symptom of atopic dermatitis.

Helping patients express concerns and how symptoms affect their daily lives is a necessity when addressing HRQL, and we believe that appropriate methods of identifying and defining pain are needed to further address this concern with patients. One approach to measuring pain is through pain-specific patient-reported outcome (PRO) measures [17]. PROs capture information of interest from the patient perspective and have demonstrated utility in identifying concerns otherwise left unexpressed in a dermatology visit [18]. These measures track patient progress and problems over time when administered regularly at visits [19], and numerous PROs currently exist for a wide range of concerns, giving clinicians many options to choose from when deciding the best measures to use with their patients. However, further studies are needed to determine which methods work best for identifying pain specific to atopic dermatitis.

### Limitations of the Study

Our study is limited in generalizability because it took place at a single academic medical center in a high-altitude, low-humidity environment conducive to increased atopic dermatitis severity. Expanding to other institutions and other geographic locations outside Utah may produce different results. Furthermore, our study only included adults, and thus our results might not be generalizable to the experiences of children and adolescents with atopic dermatitis. Although investigators used open-ended questions to capture the participants’ voices, it is possible that the way the questions were worded influenced participant responses. In using prompts to aid discussion, the interviewers may have directed a conversation toward those specifics, and some aspects were talked about more than others due to how questions were worded. This study is also limited in its interpretability because of its qualitative nature. Qualitative research provides an opportunity to learn about patient experiences and produce ideas for future research [20]. However, a small, nonrandom, single-institution cohort makes the findings from this study difficult to generalize if the data were quantified. The thematic analysis for this study does not involve quantification of themes or individual components of these themes but does provide information to help generate ideas for future studies that can appropriately quantify the phenomena presented here.

This study presents the experiences of patients and their family members in dealing with the effects of pain caused by atopic dermatitis. We found that many aspects of daily life can be affected by this symptom, and although pain can be very bothersome and significantly impact HRQL, it can be difficult for some patients to explain what they are experiencing. Clinicians must be aware that atopic dermatitis can cause pain and should ask patients about the presence and effects of pain when treating patients with atopic dermatitis.

## Abbreviations

HRQL: health-related quality of life
PRO: patient-reported outcome
